# Supplementary material for: Coiled-Coil N21 of Hpa1 in Xanthomonas oryzae pv. oryzae Promotes Plant Growth, Disease Resistance and Drought Tolerance in Non-Hosts via Eliciting HR and Regulation of Multiple Defense Response Genes
Source: Int J Mol Sci. 2020 Dec 28;22(1):203. doi: 10.3390/ijms22010203 (PMC7795061; doi:10.3390/ijms22010203)
Supplement: Supplementary file 1 [file ijms-22-00203-s001.zip › Supplementary/description.docx]

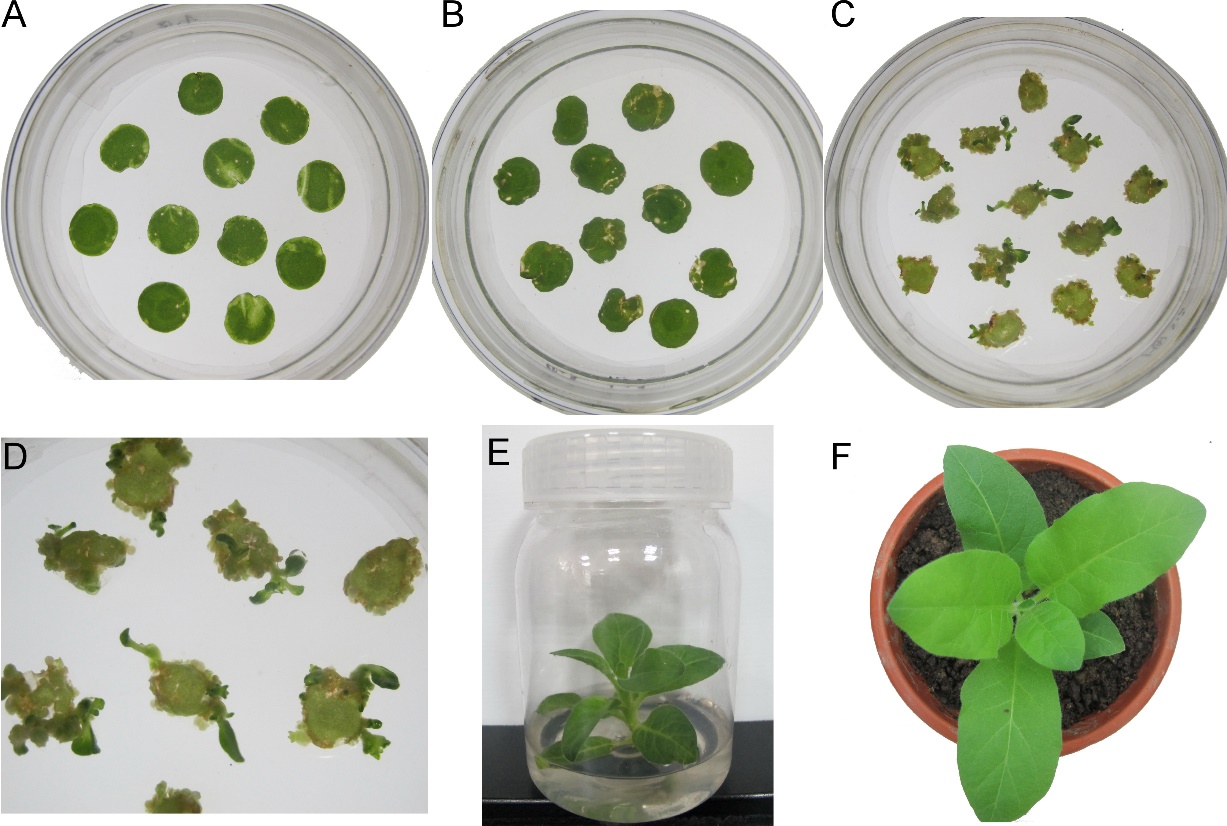


Figure S1. Process of tran-*N21* tobacco plants transformation and regeneration.

(A) The discs were first placed on a differentiation medium after the discs were co-cultivated with the *Agrobacterium*, including recombined vector pBI121-*N21*; (B) The discs were placed on a differentiation medium 2 weeks post-inoculation; (C) the formation of an initial callus on the explants at 4 weeks post-inoculation; (D) the tobacco shoots regenerated from the callus at 5 weeks post-inoculation; (E) fully differentiated transgenic tomato plants rooted in MS media with km of 100 mg/L; (F) rooted plantlets were transferred to pots.





Figure S2. PCR verification of gDNA of tran-*N21* tobacco of T3 progeny.

M, DL 2,000 DNA Marker; lines 1-7, the templates were the gDNA of different strains of tran-*N21* tobacco; line 8, the template was the recombined vector pBI121-*N21* as a positive control; line 9, the template was pBI121vector as a negative control; specific pBI121vector and 35S promoter sequences were separately used as primers in Table S1.





Figure S3. RT-PCR verification of tran-*N21* tobacco of T3 progeny.

M, DL 2,000 (the left picture) and DL15,000 (the right picture) DNA Marker; lines 1-3, tran*-N21* tobacco; line 4, tran-*pBI121* tobacco. The left was used as the internal primers of *N21*; the right was used as primers of *EF-1α* as a reference.


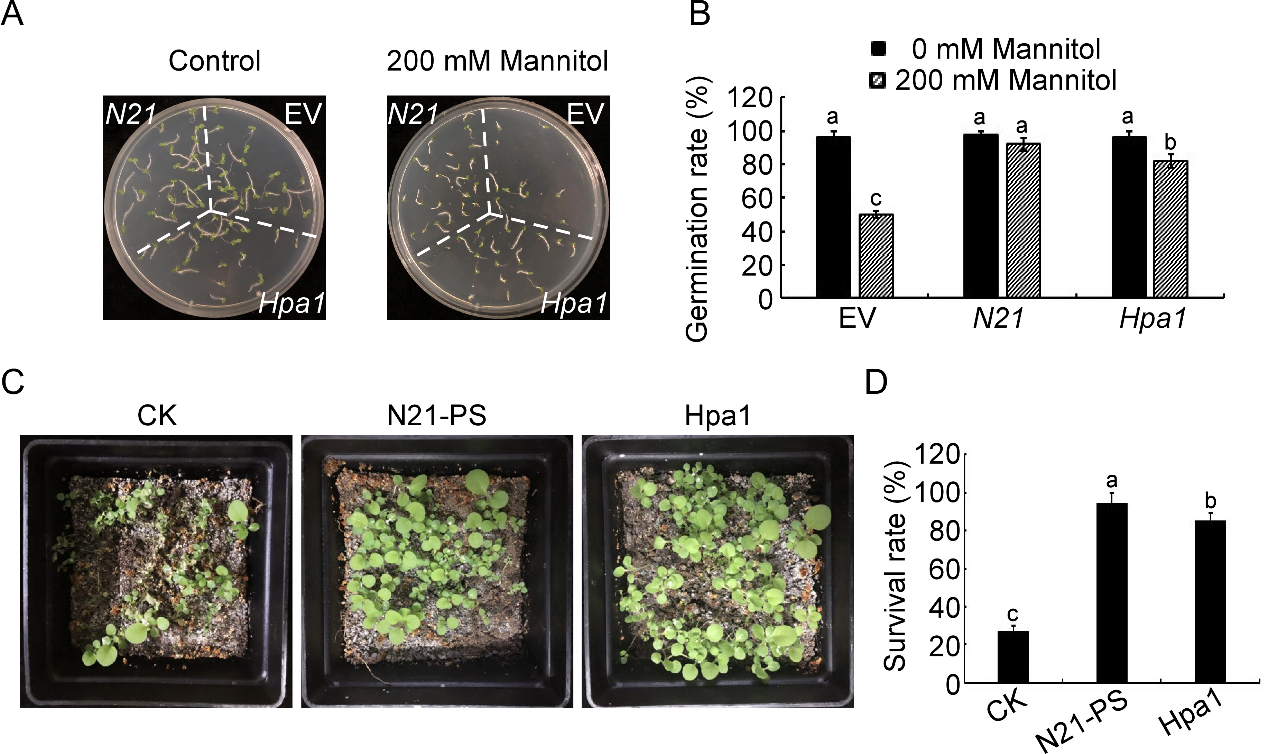


Figure S4. Seed germination and seedling survival under different treatments. (A) Seeds of EV tobacco, trans-*N21* and trans-*Hpa1* tobacco were germinated on control (0 mM mannitol) and 200 mM mannitol. Photos were taken 21 days after treatment. EV, *N21*, *Hpa1* represented transgenic empty vector tobacco, trans-*N21* tobacco and trans-*Hpa1* tobacco, separately. (B) Germination rate were measured 21 days after planting. Error bars represent the standard deviation and letters represent significant difference (Duncan’s new multiple range test, *p*<0.05). (C) 7-days seedling of Xanthi tobacco were root-irrigation with 10% PEG, then divided 3 groups: one was sprayed within 80 μg/ml N21-PS; another used the same amount of Hpa1 protein expressed from *E. coli*; the other used sterile water as negative control. The survival rate was measured 2 weeks after treatments. Error bars represent the standard deviation and letters represent significant difference (Duncan’s new multiple range test, *p*<0.05). PEG6000 represented polyethylene glycol 6000; N21-PS represented N21 peptide solution


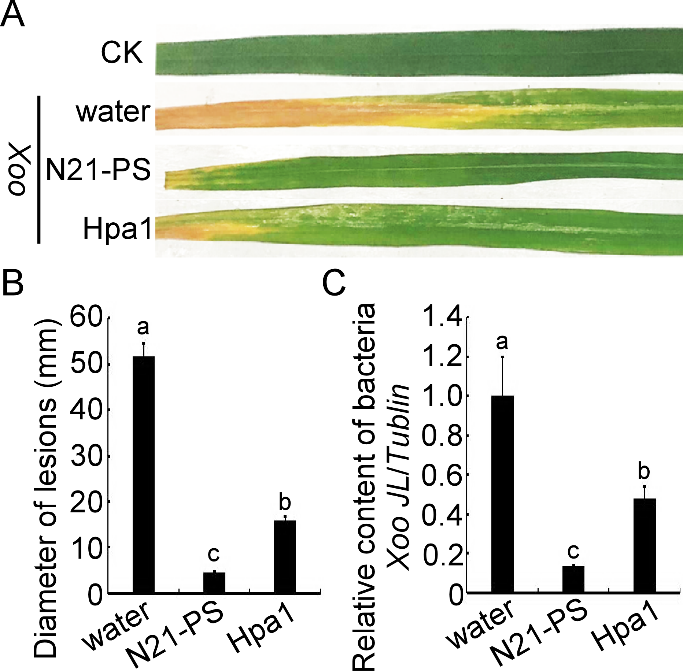


Fig. S5 N21 peptide has better resistance of rice to *Xoo* than full-length Hpa1. (A) Rice growing to tillering stage was divided into four groups, each group treated with 3 pots of rice. The first group of rice was treated with sterile water surface spray (CK). The second group of rice was inoculated with PXO99 of *Xoo* with OD600=1 through direct leaf cutting. The third and the fourth group were pre-treated with 40 μg/ml N21-PS of 3 ml or the same amount of Hpa1 protein expressed from *E. coli* for 24 hours, then inoculated the suspension of PXO99 of OD600=1 through leaf cutting and put them at 28˚C with 80% RH. Photos were taken at 10 days after inoculation. N21-PS represented N21 peptide solution. *E. coli* represented [*Escherichia*](javascript:;) [*coli*](javascript:;). RH represented relative humidity. (B) Diameter of lesions was measured at 10 days post inoculation. ±SD was calculated from three repeated experiments, and lowercase letters indicate statistically significant differences (Duncan's new multiple range test, letters mean *p*<0.05). (C) Relative content of bacteria in the diseased leaves of different treatment were measured by quantifying *Xoo JL* relative to rice *Tublin* at 10 days post inoculation. Error bars represent the standard deviation and letters represent significant difference (Duncan’s new multiple range test, *p*<0.05). *JL* is a part of *glycosyltransferase* gene sequence of *Xanthomonas*.
