# Supplementary material for: Coiled-Coil N21 of Hpa1 in Xanthomonas oryzae pv. oryzae Promotes Plant Growth, Disease Resistance and Drought Tolerance in Non-Hosts via Eliciting HR and Regulation of Multiple Defense Response Genes
Source: Int J Mol Sci. 2020 Dec 28;22(1):203. doi: 10.3390/ijms22010203 (PMC7795061; doi:10.3390/ijms22010203)
Supplement: Supplementary file 1 [file ijms-22-00203-s001.zip › Supplementary/Table S1.docx]

**Table S1. Primers used in this study**

| **Primer name** | **Sequence (5’-3’)** | **Remark** |
| --- | --- | --- |
| *CN21*-F | AGCAAGTGGATTGATGTGAT | Verification of tran-*N21* tobacco |
| *CN21*-R | CGGCGAACTGATCGTTAA | Verification of tran-*N21* tobacco |
| *35S* -F | GCCTTTTCAATTTCAGAAAGAATGC | Verification of tran-*N21* tobacco |
| *35S* -R | CGTGTTCTCTCCAAATGAAATGAAC | Verification of tran-*N21* tobacco |
| *N21*-F | ATGAACCAGGGCATCTCG | Verification of tran-*N21* tobacco |
| *N21*-R | TTAAAGCAGGGCCGAGAT | Verification of tran-*N21* tobacco |
| *EF-1α*-F | AGACCACCAAGTACTACTGCAC | Verification of tran-*N21* tobacco |
| *EF-1α*-R | CCACCAATCTTGTACACAATCC | Verification of tran-*N21* tobacco |
| Nb-RT-*EF1α*-F | ATCTCTGGTTTCGAGGGGGACA | quantitative RT-PCR analysis |
| Nb-RT-*EF1α*-R | TAGGTCCAAAGGTCACAA | quantitative RT-PCR analysis |
| Nt-RT-*PR1a*-F | ACAGGTGTGTGGACACTATAC | quantitative RT-PCR analysis |
| Nt-RT-*PR1a*-R | GAGCAATTATTTAAGTTCTTG | quantitative RT-PCR analysis |
| Nt-RT-*PR1b*-F | TTTGGATGCCCATAACACAGC | quantitative RT-PCR analysis |
| Nt-RT-*PR1b*-R | AATCGCCACTTCCCTGAGCTA | quantitative RT-PCR analysis |
| Nt-RT-*PR2*-F | CTCAACTGCAACATATTCAGGGA | quantitative RT-PCR analysis |
| Nt-RT-*PR2*-R | GTGGGACATCAGCAGTGTTGA | quantitative RT-PCR analysis |
| Nt-RT-*ACS1*-F | TGGACTTTTCTGTTGGATGGAT | quantitative RT-PCR analysis |
| Nt-RT-*ACS1*-R | TCCATTAATATTGGAGTCGAT | quantitative RT-PCR analysis |
| Nt-RT-*ACS2*-F | CCCAAAAGCCTCCATTTGCAC | quantitative RT-PCR analysis |
| Nt-RT-*ACS2*-R | CCTCCACTCATAACTATGCGA | quantitative RT-PCR analysis |
| Nt-RT-*EIN2*-F | ATTGGTCGGCAGCAAAAATCA | quantitative RT-PCR analysis |
| Nt-RT-*EIN2*-R | CGCTTGTAACGCTTTAGGACA | quantitative RT-PCR analysis |
| Nb-RT-*RBOHA*-F | GACTCGTTCCAGCGCTCATA | quantitative RT-PCR analysis |
| Nb-RT-*RBOHA*-R | TGTGCGAAATCGGAACGGTA | quantitative RT-PCR analysis |
| Nb-RT-*RBOHB*-F | TCACAAGAGCTCAGGCGTTT | quantitative RT-PCR analysis |
| Nb-RT-*RBOHB*-R | TCATCGAACCGCTTCTCGAC | quantitative RT-PCR analysis |
| Nt-RT-*LoX1*-F | CCATAGTTAC GTTGGGTAGT GA | quantitative RT-PCR analysis |
| Nt-RT-*LoX1*-R | TGATGTCAATGATGGATCTCC | quantitative RT-PCR analysis |
| Nt-RT-*Aoc4*-F | CCAATCTCTT AAACTCGGCA C | quantitative RT-PCR analysis |
| Nt-RT-*Aoc4*-R | TTGGCTCAAGCGAAGATAAGC | quantitative RT-PCR analysis |
| Nt-RT-*NPR1*-F | ATGGAAGCAA AAGTTGCAAT G | quantitative RT-PCR analysis |
| Nt-RT-*NPR1*-R | ATGATCTTATTTAGAACTTCTG | quantitative RT-PCR analysis |
| *PccRPSD*-F  *PccRPSD*-F  *GFP*-F  *GFP*-R  *NtSOD*-F  *NtSOD*-R  *NtCAT*-F  *NtCAT*-R  *NtAPX*-F  *NtAPX*-R  *NtERD10B*-F  *NtERD10B*-R  *NtLEA5*-F  *NtLEA5*-R  *NtPLC3*-F  *NtPLC3*-R  *NtCMK1*-F  *NtCMK1*-R  *OsTUBULIN*-F  *OsTUBULIN*-R  *XoJLXoo*-F  *XoJLXoo*- R | CGTCTGAAAGGGCAACACAGGTG  GCTTTTCACGCTGTTCAGCCAG  TGAGGGATACGTGCAGGAG  GGCTTTGATGCCGTTCTTT  TTTGTGTGGTGTCTTTCTTCAACA  AGCTAGTGCATGCTCCACCAT  CATGGCACTCTGCTGGTACCT  CCATTGTTTGCTCCATGTCCTT  ACTTTCCTCTTCGACGATATTGGT  TTGATTTCCCAGCCTTGTTGA  TCGCACTACGACAACCAATTTAG  GATGGACTGGGTTCCCATATTC  CGTCCCATAGCGCAATCG  GGACGCTCCATATTTTCTAACAAAC  TTATGGGTGAAGGGTGGTATTATG  GGTCGTGTAGTGAAACTGCTC  CCGTGTTACGAGCTGATCCTAA  CGGTCATTCTCTTCCTATGGTCTT  TACCGTGCCCTTACTGTTCC  CGGTGGAATGTCACAGACAC  CCTCTATGAGTCGGGAGCTG  ACACCGTGATGCAATGAAGA | quantitative RT-PCR analysis  quantitative RT-PCR analysis  quantitative RT-PCR analysis  quantitative RT-PCR analysis  quantitative RT-PCR analysis  quantitative RT-PCR analysis  quantitative RT-PCR analysis  quantitative RT-PCR analysis  quantitative RT-PCR analysis  quantitative RT-PCR analysis  quantitative RT-PCR analysis  quantitative RT-PCR analysis  quantitative RT-PCR analysis  quantitative RT-PCR analysis  quantitative RT-PCR analysis  quantitative RT-PCR analysis  quantitative RT-PCR analysis  quantitative RT-PCR analysis  quantitative RT-PCR analysis  quantitative RT-PCR analysis  quantitative RT-PCR analysis  quantitative RT-PCR analysis |
