# Supplementary figures and images for: Coiled-Coil N21 of Hpa1 in Xanthomonas oryzae pv. oryzae Promotes Plant Growth, Disease Resistance and Drought Tolerance in Non-Hosts via Eliciting HR and Regulation of Multiple Defense Response Genes
Source: Int J Mol Sci. 2020 Dec 28;22(1):203. doi: 10.3390/ijms22010203 (PMC7795061; doi:10.3390/ijms22010203)

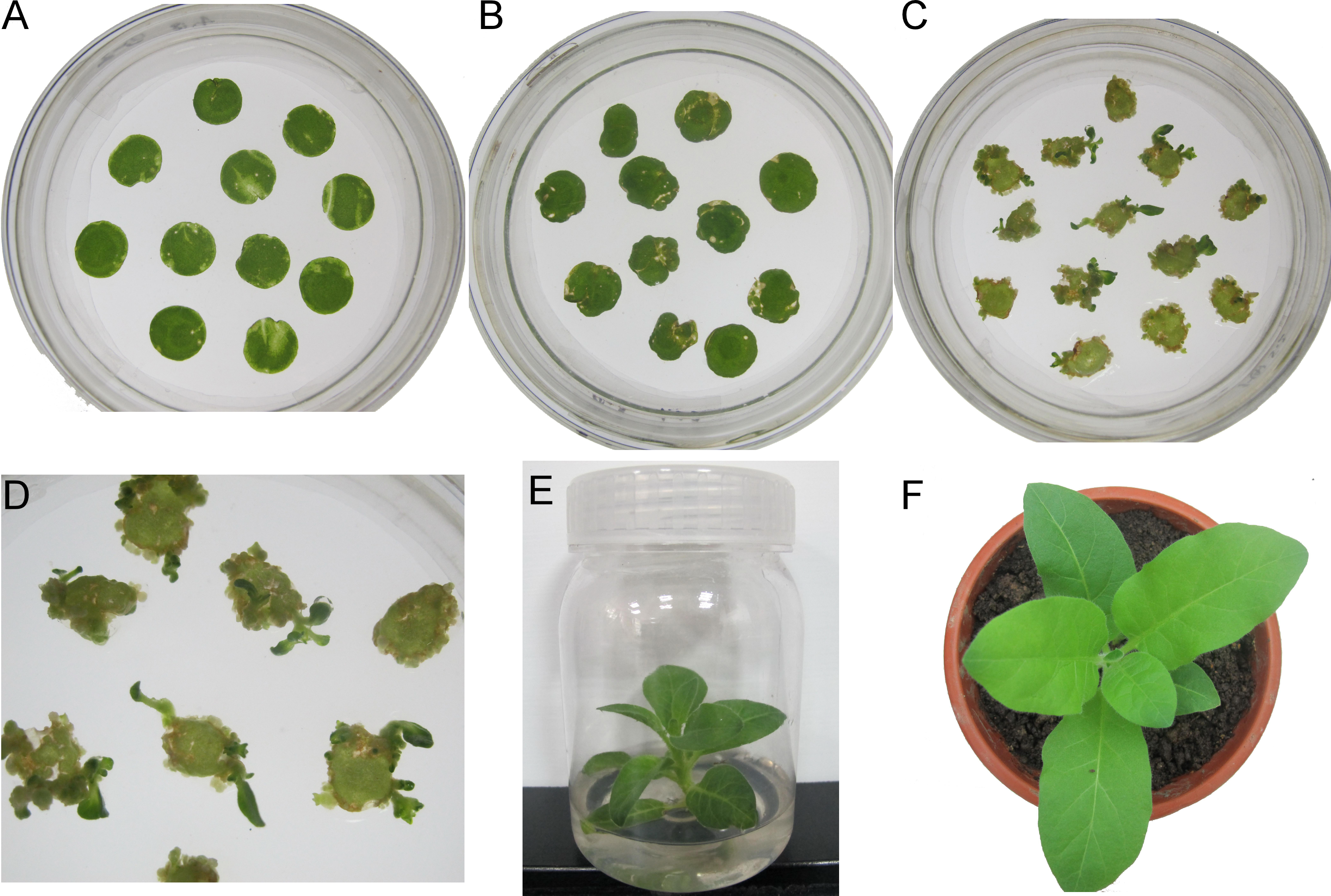

Supplement: Supplementary file 1 [file ijms-22-00203-s001.zip › Supplementary/Fig. S1.tif]

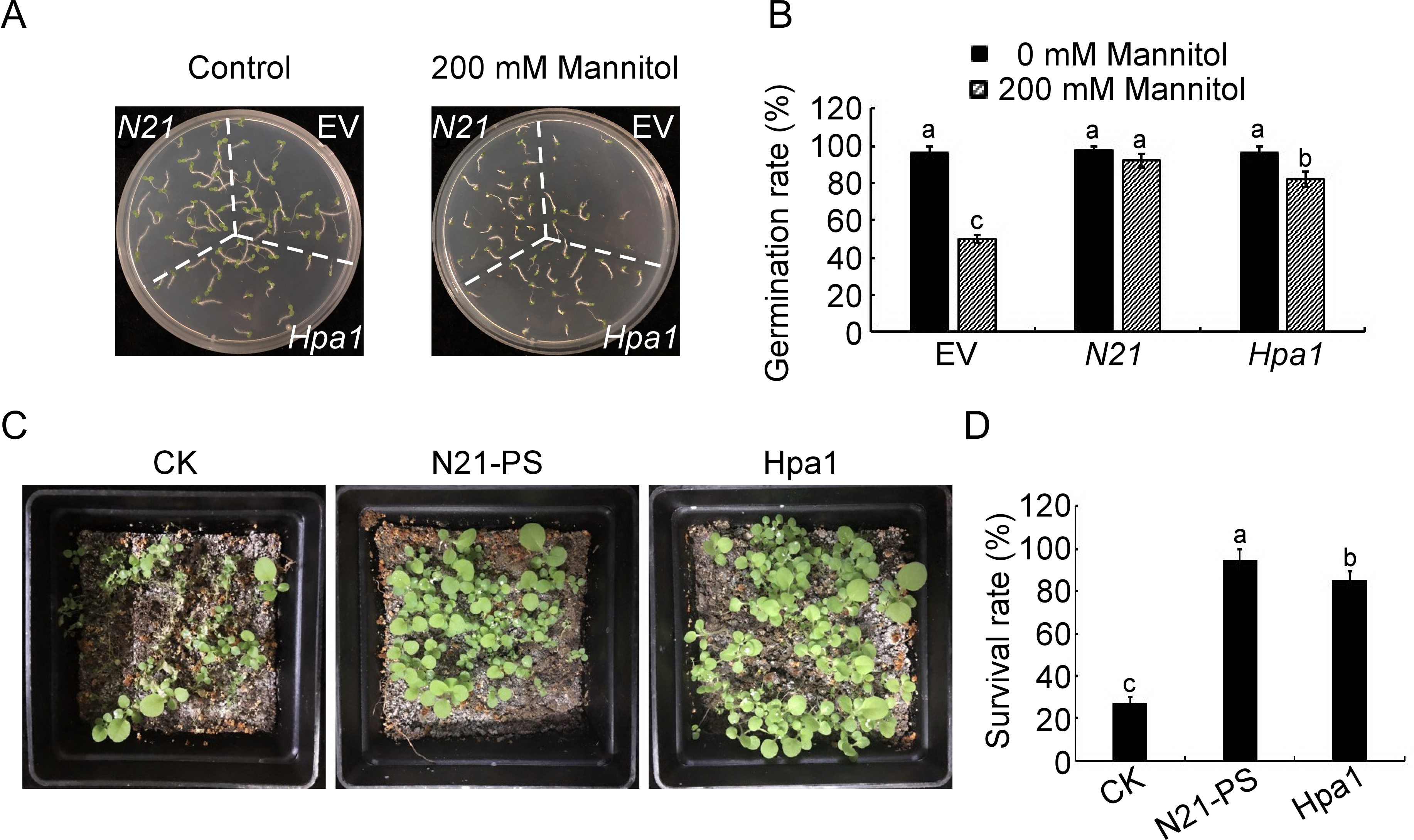

Supplement: Supplementary file 1 [file ijms-22-00203-s001.zip › Supplementary/Fig. S4.tif]

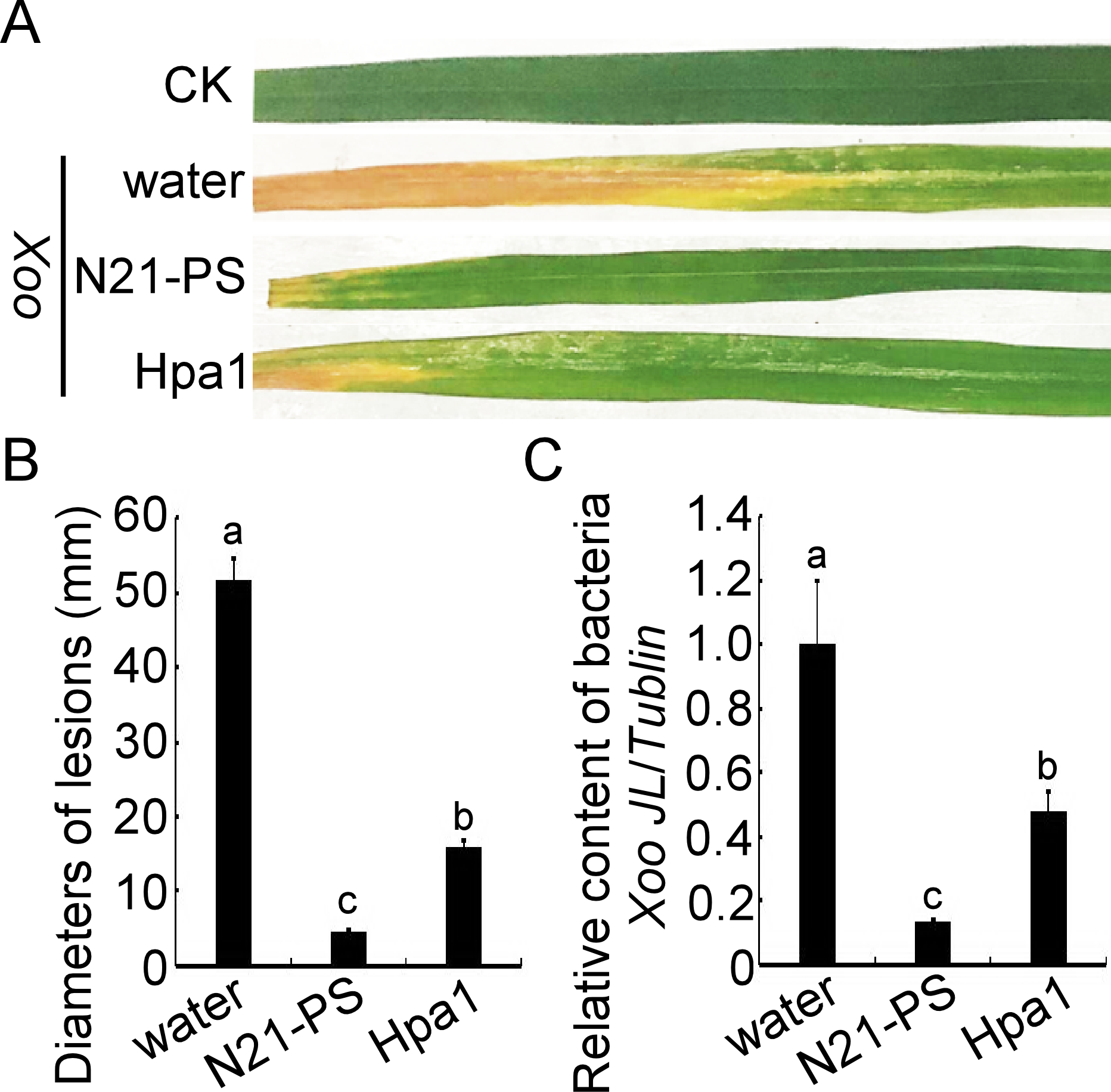

Supplement: Supplementary file 1 [file ijms-22-00203-s001.zip › Supplementary/Fig. S5.tif]
